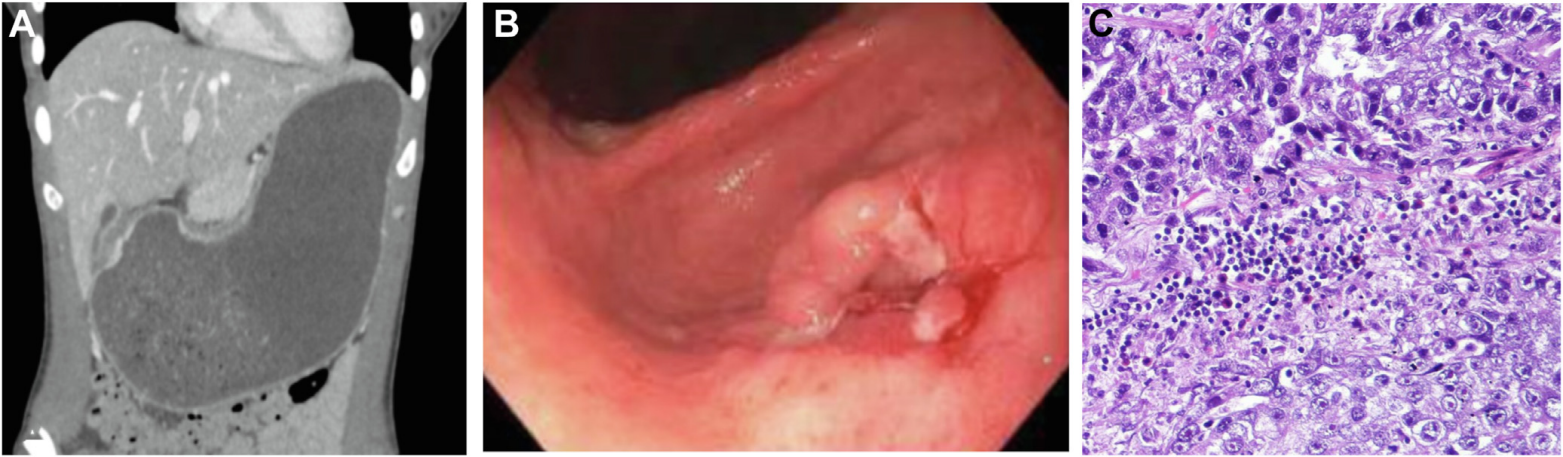# Gastric Adenocarcinoma Masquerading as Cannabis Hyperemesis Syndrome

**DOI:** 10.1016/j.gastha.2023.11.014

**Published:** 2023-12-02

**Authors:** Charles Altfillisch, Fortune Unegbu, Daniel Buckles

**Affiliations:** 1 Division of Gastroenterology and Hepatology, University of Kansas Health System, Kansas City, Kansas; 2 Department of Internal Medicine, University of Kansas Health System, Kansas City, Kansas

A 29-year-old female with a history of marijuana use presented to the Emergency Department due to intractable nausea and vomiting. Symptoms had been present for 3 months and were associated with 30-pound unintentional weight loss. She reported smoking marijuana daily. She had been to various emergency rooms on multiple occasions and was diagnosed with Cannabis Hyperemesis Syndrome.

A computed tomography scan of the abdomen revealed a severely dilated, fluid filled stomach (Figure A). A diagnostic esophagogastroduodenoscopy showed a stomach filled with a large volume of liquid that was suctioned and an ulcerated, friable mass with spontaneous bleeding in the prepyloric region (Figure B) causing gastric outlet obstruction. Biopsies from the mass were positive for invasive adenocarcinoma (Figure C). The patient underwent a partial gastrectomy with Roux-en-Y reconstruction and D2 lymphadenectomy.

While Cannabis Hyperemesis Syndrome is common, this case underscores the importance of a complete diagnostic evaluation to exclude other pathology, especially when red flag symptoms, such as weight loss, are present. The average age of diagnosis of gastric adenocarcinoma is 68 and less than 10% of cases are in patients younger than 40. Given the increasing prevalence with advancing age, the rarity in such a young patient may have additionally contributed to the delay in diagnosis.

**Figure:**